# Recurrent Cardiac Arrests Due to Amniotic Fluid Embolism

**DOI:** 10.7759/cureus.22475

**Published:** 2022-02-21

**Authors:** Munsef Barakat, Ans Alamami, Ali Ait Hssain

**Affiliations:** 1 Internal Medicine Residency Program, Medical Education, Hamad General Hospital, Doha, QAT; 2 Critical Care, Hamad General Hospital, Doha, QAT; 3 Anesthesiology/Critical Care, Hamad General Hospital, Doha, QAT

**Keywords:** retrievable inferior vena cava filter, post partum haemmorrhage, pulmonary embolism (pe), leading cardiac arrest, disseminated intravascular coagulation (dic), afe (amniotic fluid embolism)

## Abstract

Amniotic fluid embolism (AFE) is a rare but devastating complication of pregnancy and is associated with high morbidity and mortality. Identifiable maternal risk factors for AFE include older age, multiparity, cesarean section, and placenta previa, while fetal factors include male gender, fetal distress, and death. AFE presents with respiratory distress, seizure, and circulatory collapse and can be complicated with disseminated intravascular coagulopathy, multiorgan failure, and death. In our case, we present a patient who underwent elective cesarean section for placenta previa, which was complicated by sudden cardiac arrest immediately after delivering the placenta in the operating theatre followed by disseminated intravascular coagulation (DIC). The patient developed massive post-partum hemorrhage secondary to the underlying DIC, which required a massive blood transfusion along with platelets, fresh frozen plasma (FFP), and tranexamic acid. The Society of Maternal-Fetal Medicine proposed criteria for the diagnosis of AFE, which include clinical features and laboratory findings. The presence of a DIC picture is considered to be the hallmark finding that helps to differentiate between AFE and other conditions with similar presentation. Treatment of amniotic fluid embolism depends on early recognition and supportive care.

## Introduction

Amniotic fluid embolism (AFE) is a rare but devastating complication of pregnancy and is associated with high morbidity and mortality. Fitzpatrick et al. estimated that the incidence of AFE ranged from 0.8 to 1.8 per 100,000 maternities, and 30% to 41% of women subject to AFE died or had a permanent neurological injury [1].

Other reported incidences of AFE ranged from 1.9 cases per 100,000 maternities (UK) to 6.1 per 100,000 maternities (Australia) [2]. AFE presents with sudden respiratory distress, seizure, and circulatory collapse and can be complicated with disseminated intravascular coagulopathy and multiorgan failure.

Identifiable maternal risk factors for AFE include older age, multiparity, cesarean section, and placenta previa, while fetal factors include male gender, fetal distress, and death. The pathophysiology of AFE is still poorly understood. Nevertheless, prompt suspicion, diagnosis, and resuscitation are needed to optimize the outcome. We present a young patient who developed a cardiac arrest due to AFE immediately after an elective cesarean section for placenta previa; her case was complicated by coagulopathy and acute kidney injury (AKI).

## Case presentation

A 34-year-old woman, gravida 3, para 2, underwent an elective cesarean section for placenta previa grade III at 37 weeks of gestation. The surgery was performed under combined spinal and epidural anesthesia at the level of L3-L4, with 12.5 mg of bupivacaine 0.5% with fentanyl 10 mcg administered intrathecally. The surgery started at 11:32 AM, a lower segment cesarean section was performed, and a male infant was delivered at 11:42 AM with an APGAR score of 9 and 10 at one and five minutes. 

Following the delivery of the placenta via continuous cord traction, at 11:44 AM, the patient suddenly became pale and developed a tonic-clonic seizure followed by pulseless electrical activity (PEA) arrest. Cardiopulmonary resuscitation (CPR) was started immediately, and the surgical wound was closed; meanwhile, the CPR was ongoing. CPR continued for three cycles, for which a total of 3 mg of adrenaline and fluid resuscitation were administered. A right central venous catheter was inserted, and noradrenaline was started after achieving the return of spontaneous circulation (ROSC) due to hypotension. 

**Figure 1:**
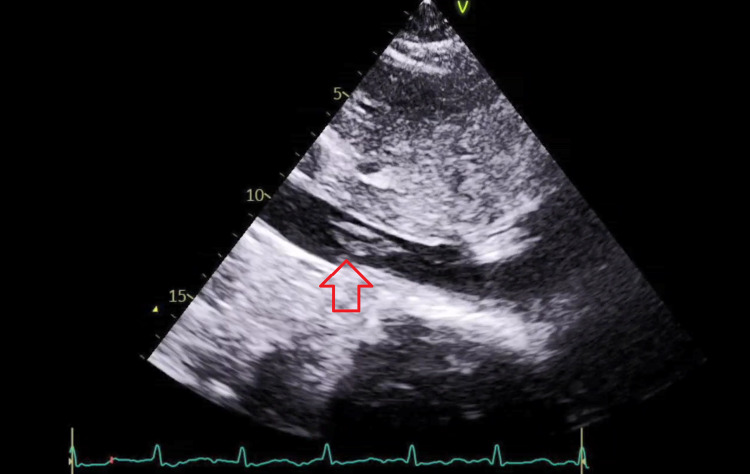
Bedside echocardiography showing IVC thrombus

**Figure 2:**
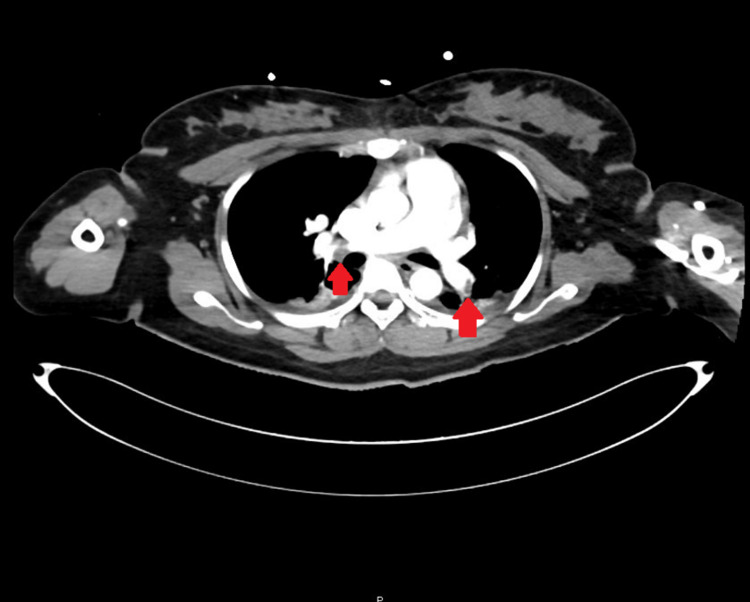
CT pulmonary angiography showing bilateral pulmonary embolism

The patient continued to have uterine bleeding with an estimated blood loss of 2.7 L, and an intrauterine (Bakri®) balloon was placed by the obstetric team in an attempt to control the bleeding. Rotation thromboelastometry (ROTEM) showed profound disseminated intravascular coagulation (DIC) with a straight line in the FIBTEM A5 component of the ROTEM. Shortly after the hemoglobin (Hb) dropped and the massive blood transfusion protocol was activated, the patient received an initial of 6 units of packed red blood cells (RBCs), 4 units of fresh frozen plasma (FFP), 6 units of platelets, 2 grams of tranexamic acid, 10 grams of fibrinogen, and cryoprecipitate. Blood gas analysis indicated lactic acidosis with lactate of 7. A clinical diagnosis of AFE was made and the patient was transferred to the intensive care unit (ICU) where a hemodynamic assessment was performed using the Pulse Contour Cardiac Output 9PiCCO0 study and the patient was kept on noradrenaline, vasopressin, and continued supportive care with blood product transfusions as needed (Table 1). She developed AKI but continued to produce a good amount of urine. The patient developed an episode of hyperkalemia that was managed medically. Echocardiography showed dilated RV and RV dysfunction with a tricuspid annular plane systolic excursion (TAPSE) of 1.7 cm and an RVSP of 51 mmgh along with evidence of IVC mobile thrombus (Figure 1). CT pulmonary angiography and abdominal CT with contrast were done to show filling defects in the distal segments of the bilateral main pulmonary arteries extending to the upper and lower lobe pulmonary artery divisions in keeping with pulmonary embolism (Figure 2). The pulmonary trunk was mildly prominent and measured up to 30 mm in diameter. The left ovarian vein is significantly engorged, demonstrating a large filling defect within, suggestive of an embolus. As Hb continued to drop, a conventional angiogram for both internal iliac arteries was performed, and it did not show any active extravasation. Based on that, a decision was made against embolization, and to keep up the conservative measures, the obstetric team kept the patient on an oxytocin infusion for 24 hours. Uterine bleeding improved, and the Bakri balloon was eventually removed. Subsequently, the intensive care team was able to titrate down and eventually stop the ionotropic support. Once the patient was in DIC and given her ovarian vein thrombus, an IVC filter was inserted to prevent emboli showering, and once the patient was out of DIC, a heparin infusion was initiated. The patient was successfully extubated on the third postoperative day with no neurological sequelae; the coagulopathy improved, and the platelets stabilized.

**Table 1:** PiCCO study reports PiCCO: Pulse index Contour Continuous Cardiac Output, CO: cardiac output, SV: stoke volume, HR: heart rate, SBP: systolic blood pressure, DBP: diastolic blood pressure, MAP: mean arterial pressure, CI: cardiac index, SVR: systemic vascular resistance, CVP: central venous pressure, GEDVI: global end diastolic volume index, EVLW: extravascular lung water, ITBVI: intrathoracic blood volume index.

	On the day of admission	3^rd^ day of admission
CO	4.2 L/min	5.56 L/min
SV	38 ml	46.7 ml
HR	110 bpm	121 bpm
SBP	91 mmHg	158 mmHg
DBP	44 mmHg	69 mmHg
MAP	59 mmHg	88 mmHg
CI	2.8 L/min/m^2^	3.27 L/min/m^2^
SVR	1577 dynes-sec/cm^5^	1738 dynes-sec/cm 5
CVP	23 cmH_2_O	17 cmH_2_O
GEDVI	802 ml/m^2^	874 ml/m^2^
EVLW	21 mL/kg	17.4 mL/kg
ITBVI	1201 ml/m^2^	714 ml/m^2^

## Discussion

In our case report, we presented a patient who underwent elective cesarean section for placenta previa. Unfortunately, she developed a sudden cardiac arrest immediately after delivering the placenta to the operating theatre. Immediate labs collected showed a DIC picture.

The patient developed massive post-partum hemorrhage due to her DIC, which required a massive blood transfusion along with platelets, FFP, and tranexamic acid. A Bakri intrauterine balloon was inserted and started on an oxytocin infusion to control the bleeding to avoid hysterectomy, which eventually was the right decision.

After stabilization, CT pulmonary angiography revealed bilateral pulmonary embolism. As the patient was in DIC, anti-coagulation was not started. By that time, the patient was on multiple vasopressors and her hemoglobin continued to drop despite multiple blood transfusions, so abdominal CT angiography was arranged, which excluded any active bleeding but revealed a right ovarian vein thrombus. An IVC filter was inserted to prevent emboli showering. Once the patient was out of DIC, heparin infusion was started.

The patient was managed with cardiorespiratory support and transfusion of blood products as needed. Eventually, she was stabilized and extubated successfully without any neurological or cardiac sequelae.

The pathophysiology of AFE is not completely understood. The initial theory was that the amniotic fluid enters the maternal circulation, obstructing the pulmonary circulation. A recent and popular hypothesis is that a breach in maternal-fetal circulation allows the entry of amniotic fluid (which contains fetal cells and fetal antigenic material) into the maternal systemic circulation, causing an abnormal and exaggerated immune response. The role of complement system activation in this immune response has been described by Benson [3] (in Japan, the registry program for AFE). The analysis of the data showed that there were two types of AFE: type I, present with circulatory collapse due to mechanical obstruction of the pulmonary circulation by the fetal materials; and type II, which is caused by an anaphylactoid-like reaction to fetal materials, which leads to pulmonary vasospasm and activation of platelets, white blood cells, and the complement system and may lead to DIC and atonic uterine bleeding [4].

The first published description of AFE was by Steiner, who reported a series of cases of sudden peripartum deaths in which postmortem examination of the maternal pulmonary circulation revealed the presence of amniotic fluid material [5]. Although the original theory of mechanical obstruction of the pulmonary circulation remained valid for many years, recently, more evidence became available suggesting that the pathogenesis underlying the AFE is more complicated than the original theory had assumed. Evidence suggests that once a maternal-fetal circulation breach occurs and amniotic/fetal material enters the maternal systemic circulation, it causes a systemic inflammatory response syndrome through activation of proinflammatory mediators, eventually leading to shock and activation of the coagulation cascade, leading to DIC [6-8]. 

In a trial done in 1968, human amniotic fluid was injected into rabbits. In this trial, it was noticed that the severity of the symptoms was related to the number of cells injected. This study estimated that the amount of amniotic fluid needed for a woman to develop severe AFE symptoms is around 7 L [9]. An animal study found that pretreatment with a leukotriene synthesis inhibitor before injecting amniotic fluid emboli into the animal subject prevented death in the model [10].

The Society of Maternal-Fetal Medicine proposed criteria for diagnosis of AFE (Table 2) which includes clinical features and laboratory findings [11]. The presence of DIC is the hallmark finding that helps to differentiate between AFE and other conditions with similar presentation. The International Society on Thrombosis and Hemostasis (ISTH), modified for pregnancy scoring system (Table 2) has been validated and is used to detect DIC in pregnancy.

**Table 2:** Maternal-fetal medicine proposed criteria for diagnosis of AFE

Maternal-fetal medicine proposed criteria for diagnosis of AFE (all criteria must be present)
Sudden cardiorespiratory arrest or hypotension and de-saturation
Documented DIC
No fever during labor
Clinical onset during labor or within 30 minutes of placenta delivery

Treatment of AFE is supportive, including the prompt exclusion of other correctable causes. Cardiopulmonary resuscitation and volume replacement along with the use of vasopressors if needed. Hypoxia and respiratory arrest should be treated, and most patients require intubation and mechanical ventilation, while ECMO (extracorporeal membrane oxygenation) has been used as well in a few cases. Coagulopathy needs to be corrected with transfusion of blood products as needed. Delivery of the fetus and placenta must be carried out promptly.

## Conclusions

AFE is a rare but unfortunate and lethal complication of pregnancy. Although a proven diagnosis is not needed for treatment, other correctable causes need to be excluded. In our patient, we argue that the diagnosis is AFE rather than PE, as this patient developed sudden cardiac arrest immediately after delivering the placenta and the ROTEM showed a picture of DIC which was complicated by post-partum hemorrhage. Later, she developed hypercoagulable status, including PE and ovarian vein thrombosis. With supportive care and the insertion of an IVC filter to prevent further showering of any emboli, the patient's condition stabilized and was extubated without any neurological complications.
